# Supplementary material for: Role and mechanism of NCAPD3 in promoting malignant behaviors in gastric cancer
Source: Front Pharmacol. 2024 Apr 22;15:1341039. doi: 10.3389/fphar.2024.1341039 (PMC11070777; doi:10.3389/fphar.2024.1341039)
Supplement: Supplementary file 11 [file DataSheet2.ZIP › GSEA/Oncogenic signature/my_analysis.Gsea.1599461962066/RPS14_DN.V1_UP.html]

Details for gene set RPS14\_DN.V1\_UP[GSEA]

|  || Dataset | filtered\_dataset.sample\_info.cls#WT\_versus\_NCAPD3\_MUT |
| Phenotype | sample\_info.cls#WT\_versus\_NCAPD3\_MUT |
| Upregulated in class | NCAPD3\_MUT |
| GeneSet | RPS14\_DN.V1\_UP |
| Enrichment Score (ES) | -0.46230698 |
| Normalized Enrichment Score (NES) | -1.9168297 |
| Nominal p-value | 0.004761905 |
| FDR q-value | 0.031873215 |
| FWER p-Value | 0.232 |
Table: GSEA Results Summary

  

Fig 1: Enrichment plot: RPS14\_DN.V1\_UP      
 Profile of the Running ES Score & Positions of GeneSet Members on the Rank Ordered List

  

| SYMBOL | TITLE | RANK IN GENE LIST | RANK METRIC SCORE | RUNNING ES | CORE ENRICHMENT || 1 | 57103 | TIGAR | 37 | 0.963 | 0.0870 | No |
| 2 | 64866 | CDCP1 | 515 | 0.466 | -0.1997 | No |
| 3 | 6385 | SDC4 | 871 | -0.282 | -0.4208 | Yes |
| 4 | 4088 | SMAD3 | 930 | -0.353 | -0.4207 | Yes |
| 5 | 4680 | CEACAM6 | 955 | -0.374 | -0.3938 | Yes |
| 6 | 10123 | ARL4C | 957 | -0.377 | -0.3501 | Yes |
| 7 | 3556 | IL1RAP | 1099 | -0.476 | -0.3950 | Yes |
| 8 | 10681 | GNB5 | 1105 | -0.481 | -0.3418 | Yes |
| 9 | 2012 | EMP1 | 1119 | -0.494 | -0.2929 | Yes |
| 10 | 604 | BCL6 | 1136 | -0.504 | -0.2449 | Yes |
| 11 | 5154 | PDGFA | 1191 | -0.566 | -0.2169 | Yes |
| 12 | 330 | BIRC3 | 1224 | -0.600 | -0.1691 | Yes |
| 13 | 26999 | CYFIP2 | 1234 | -0.611 | -0.1036 | Yes |
| 14 | 55521 | TRIM36 | 1337 | -0.774 | -0.0854 | Yes |
| 15 | 1475 | CSTA | 1400 | -1.162 | 0.0072 | Yes |
Table: GSEA details [plain text format]

  

Fig 2: RPS14\_DN.V1\_UP      
 Blue-Pink O' Gram in the Space of the Analyzed GeneSet

  

Fig 3: RPS14\_DN.V1\_UP: Random ES distribution      
 Gene set null distribution of ES for **RPS14\_DN.V1\_UP**
